# Beyond the promise: evaluating and mitigating off-target effects in CRISPR gene editing for safer therapeutics

**DOI:** 10.3389/fbioe.2023.1339189

**Published:** 2024-01-18

**Authors:** Rui Lopes, Megana K. Prasad

**Affiliations:** Roche Pharma Research and Early Development, Pharmaceutical Sciences, Roche Innovation Centre Basel, F Hoffmann-La Roche Ltd., Basel, Switzerland

**Keywords:** gene editing, CRISPR-Cas, off-target activity, safety, pre-clinical development, regulatory guideline, health authority

## Abstract

Over the last decade, CRISPR has revolutionized drug development due to its potential to cure genetic diseases that currently do not have any treatment. CRISPR was adapted from bacteria for gene editing in human cells in 2012 and, remarkably, only 11 years later has seen it’s very first approval as a medicine for the treatment of sickle cell disease and transfusion-dependent beta-thalassemia. However, the application of CRISPR systems is associated with unintended off-target and on-target alterations (including small indels, and structural variations such as translocations, inversions and large deletions), which are a source of risk for patients and a vital concern for the development of safe therapies. In recent years, a wide range of methods has been developed to detect unwanted effects of CRISPR-Cas nuclease activity. In this review, we summarize the different methods for off-target assessment, discuss their strengths and limitations, and highlight strategies to improve the safety of CRISPR systems. Finally, we discuss their relevance and application for the pre-clinical risk assessment of CRISPR therapeutics within the current regulatory context.

## 1 Introduction

Gene editing holds great promise in treating genetic disorders that currently have no treatment available. The development of gene editing tools such as zinc finger nucleases (ZFN), transcription activator-like effector nucleases (TALENs), meganucleases and Clustered Regularly Interspaced Short Palindromic Repeat (CRISPR)-Cas systems has enabled researchers to target previously inaccessible regions of the genome. Over the last decade, CRISPR-Cas has emerged as the front runner among other gene editing modalities for use in therapeutic molecules. Indeed, at the time of revising this article, the first CRISPR-based medicine—exagamglogene autotemcel (exa-cel), an ex *vivo* gene edited cell therapy developed by Vertex Pharmaceuticals and CRISPR Therapeutics—had received approval for treating patients with sickle cell disease and transfusion-dependent beta-thalassemia. The success of CRISPR-Cas is linked to its relatively high efficiency in generating a variety of genomic edits, the cost-effective design of single guide RNA (sgRNA) and the ease of programming this system (Hsu et al., 2014). The most popular CRISPR system consists of the Cas9-sgRNA complex, which is able to generate specific DNA double-strand breaks (DSBs) at the target location adjacent to a protospacer-adjacent motif (PAM) (Jinek et al., 2012). This results in the activation of homology-directed repair (HDR) or non-homologous end joining (NHEJ) pathways, allowing for genome editing (Hsu et al., 2013; Mali et al., 2013). Further engineering of the Cas9 protein has enabled DSB-independent editing mechanisms, such as the use of catalytically inactive Cas9 (dCas9) in combination with effector domains to activate/repress gene expression, the use of Cas9 nickases coupled to nucleobase deaminases to produce specific nucleotide alterations via strand-biased DNA repair (base editors) and the use of Cas9 nickase coupled to a reverse transcriptase enzyme to insert sequences of interest (prime editors).

One of the major concerns in the development of CRISPR-based therapeutics is the specificity of CRISPR systems. This concern stems from the well-documented evidence that CRISPR-Cas is prone to cause unwanted DNA alterations (Hsu et al., 2013; Mali et al., 2013). These undesirable effects may be the result of on-target or off-target alterations that lead to small insertions and deletions (indels) or large structural variations (SVs). Therefore, it is critical to assess the genome-wide specificity of CRISPR tools and ensure that they are safe for therapeutic applications. In this article, we provide an overview of the methods that have been used to identify and characterize on-target and off-target effects caused by CRISPR systems. We discuss their strengths and limitations, and highlight strategies to improve the safety of CRISPR systems. Finally, we discuss their relevance and application for the pre-clinical risk assessment of CRISPR therapeutics within the current regulatory framework of the United States Food and Drug Administration (FDA) and the European Medicines Agency (EMA).

## 2 Assessment of CRISPR-induced off-target editing

### 2.1 Prediction of off-targets using *in silico* methods

Off-target effects can be minimized effectively through the *in silico* prediction of CRISPR-Cas cleavage specificity and the strategic design of optimal sgRNAs. Two primary methods have been developed for predicting the specificity of CRISPR sgRNAs (Table 1). The first are alignment-based methods that employ either conventional or specialized algorithms to align sgRNAs with a given genome. As a result of this alignment, potential off-target sites and sequences are identified. This approach is mainly utilized for screening sgRNA designs and identifying all potential off-targets. The second are scoring-based methods that use complex models to score and rank sgRNAs based on the off-targets identified through the alignment process. The goal of scoring-based methods is selecting the sgRNA with the highest specificity for experimental use. Among these tools, we highlight Cas-OFFinder (Bae et al., 2014) since it is one of the most popular tools for identifying potential off-target sites. Importantly, *in silico* methods do not account for the intricate cellular environment and, therefore, their predictions need to be validated experimentally. For additional details, we refer to comprehensive reviews on this topic (Bao et al., 2021).

**TABLE 1:** In silico tools for off-target prediction.

Tool	Method	Advantages	Disadvantages
CasOT Xiao et al. (2014)	Alignment	User-defined PAM and number of mismatches	Slow speed output. Bulges are not allowed
Cas-OFFinder Bae et al. (2014)	Alignment	User-defined PAM, sgRNA length and number of mismatches. Allows bulges	Moderate speed output
FlashFry McKenna and Shendure, (2018)	Alignment	User-defined PAM and number of mismatches. High speed output. Suitable for large datasets	Bulges are not allowed
Crisflash Jacquin et al. (2019)	Alignment	User-defined PAM and number of mismatches. High speed output	Bulges are not allowed
MIT Hsu et al. (2013)	Scoring	Good ranking performance. Web platform, implemented in the CRISPOR website Haeussler et al. (2016)	Scorings are outperformed by more recent tools. Bulges are not allowed
CROP-IT Singh et al. (2015)	Scoring	Web platform. Good ranking performance Haeussler et al. (2016)	Scorings are outperformed by more recent tools
CFD Doench et al. (2016)	Scoring	Based on experimental datasets. Very good ranking performance Haeussler et al. (2016)	Requires command line
DeepCRISPR Chuai et al. (2018)	Scoring	Based on experimental datasets. Very good ranking performance. Includes both sequence and epigenetic features	Requires command line. On-target training data may contain noise. Does not take into account indels relative to the sgRNA target sequence

### 2.2 Detection of off-targets using experimental methods

Several experimental methods have been developed to identify off-target (and on-target) edits induced by CRISPR systems. These can be broadly divided into *in vitro* cell-free methods and cell-based methods. More recently, methods have also been developed that can be applied *in vivo* in pre-clinical animal studies. Table 2 summarizes the most commonly used methods for off-target assessment as well as some relatively new methods that enable *in vivo* analyses. Cell-free methods such as CIRCLE-seq or SITE-seq, which are based on the use of isolated genomic DNA (gDNA), tend to be more sensitive than cell-based methods such as TTISS or IDLV and allow easy assessment of dose response. However, *in vitro* cell-free methods are prone to lower validation rates than cell-based methods due to the lack of chromatin context in gDNA. Indeed, chromatin structure has been shown to influence CRISPR off-targeting (Kuscu et al., 2014). Furthermore, some methods such as CHANGE-seq or GUIDE-seq are “unbiased” in that they can identify genome-wide off-targets, whereas others such as UdiTas or LAM-HTGTS require *a priori* knowledge of off-target sites (from *in silico* predictions for instance). However, these latter two techniques have the added advantage of being able to identify SVs in addition to indels induced by Cas nucleases. For detailed descriptions and comparisons of the different methods, we invite the reader to refer to Table 2 and some recent reviews (Kim et al., 2019; Atkins et al., 2021; Guo et al., 2023). Each method has its own set of advantages and limitations; hence, no single method can provide a comprehensive assessment of CRISPR-Cas associated off-targets. Developers of CRISPR therapeutics will have to use a combination of methods to assess off-target editing activity of their molecules. We further discuss off-target assessment strategies for the pre-clinical development of CRISPR therapeutics in Section 4.

**TABLE 2:** Experimental methods for the detection of off-target editing by CRISPR-Cas9.

Category	Method	Description	Advantages	Disadvantages
*in vitro*	Digenome-seq Kim et al. (2015)	gDNA is digested with RNPs and subjected to WGS. Cut sites are identified bioinformatically as sites that share the exact same sequence at one end of the sequencing read	High sensitivity	High false positive rate due to lack of chromatin accessibility context, expensive due to reliance on WGS
	DIG-seq Kim and Kim, (2018)	Cell-free chromatin is subjected to Digenome-seq	Accounts for the chromatin context and hence has a higher validation rate than Digenome-seq	Relatively expensive due to the continued dependence on WGS
	nDigenome-seq Kim et al. (2020)	gDNA is digested by Cas9 nickase followed by WGS. Nick sites are identified as sites with both staggered and straight read alignments	High sensitivity, genome-wide profiling of DNA SSBs induced by nickases	Lacking in cellular context. Indirect method for profiling the genome-wide specificity of prime and base editors using Cas9 nickases
	Extru-seq Kwon et al. (2023)	Cells are pre-incubated with RNPs, then passed through an extruder to lyse the cells and bring the RNPs and gDNA in proximity. Unrepaired cut sites are identified by WGS and the Digenome-seq algorithm	High validation rate, easily adaptable to different primary cells	Difficult to identify SVs, costly
	SITE-seq Cameron et al. (2017)	gDNA is digested with RNPs and cut sites are labelled with biotinylated primers and enriched using streptavidin beads and sequenced. Cut sites are identified by read pileup	Less expensive as enrichment strategy enables shallower sequencing (∼0.62–2.46 million reads)	Low validation rate due to lack of chromatin context
	EndoV-seq Liang et al. (2019)	*In vitro* cleavage of inosine, the nucleoside intermediate that is created by ABEs, by endonuclease V (EndoV) followed by WGS	Sensitivity comparable to Digenome-seq. Multiplexed analysis of ABE-sgRNA complexes	Lacks nuclear/chromatin context Analysis limited to ABEs
	CIRCLE-seq Tsai et al. (2017)	gDNA is fragmented by sonication, circularized, and incubated with RNPs. Only circles containing nuclease digestion sites are linearized and used to create a sequencing library	Less expensive as enrichment strategy enables shallower sequencing (4–5 million reads)	Very high input requirement (∼25 μg DNA)
	CHANGE-seq Lazzarotto et al. (2020)	Similar to CIRCLE-seq, but uses enzymatic fragmentation instead of sonication to fragment gDNA	Lower input requirement than CIRCLE-seq	Lack of chromatin context
	UDiTaS Giannoukos et al. (2018)	Detects DSBs by using universal adapters and anchored primers to analyze repair outcomes after nuclease cleavage	Can detect translocations, inversions, and large deletions using short-read sequencing	Requires *a priori* knowledge for target enrichment
Cell-based	Whole Genome Sequencing (WGS) Smith et al. (2014), Veres et al. (2014), Iyer et al. (2015)	WGS on DNA extracted from cells treated with Cas9 and sgRNA	Detects several types of off-target edits including INDELs and SVs	Poor signal to noise ratio, limited sensitivity for rare variants, expensive due to need for high coverage (20–60X)
	Integrase-Defective Lentiviral vector (IDLV) integration Gabriel et al. (2011), Wang et al. (2015)	Cells are transfected with Cas9 and sgRNA plasmids and transduced with an IDLV with a propensity to integrate near DSBs, tagging the nuclease generated cut sites with lentiviral sequences. The IDLV integrated sites are then enriched via linear amplification-mediated (LAM) PCR or non-restrictive LAM PCR using primers complementary to the IDLV sequences, followed by NGS	Applicable for a variety of nuclease platforms and cell types	Lower sensitivity (0.5%) and high false positive rate
	GUIDE-seq Tsai et al. (2015)	Enriches nuclease-induced DSBs by the insertion of a double stranded oligonucleotide (dsODN) with a known sequence. dsODN specific primers are used for enrichment followed by sequencing	High validation rate and high sensitivity, commonly used	dsODN are cytotoxic for some cell lines, this approach is not feasible *in vivo*, cannot detect SVs
	iGUIDE Nobles et al. (2019)	GUIDE-seq protocol with a longer dsODN and dedicated software package	Enables detection of mispriming events lowering the false positive rate	Not commonly used
	Tagmentation-based tag integration site sequencing (TTISS) Schmid-Burgk et al. (2020)	Similar to GUIDE-seq, but uses tagmentation to shear cell-derived gDNA and tag it with Illumina sequencing adaptors	Enables multiplexed screening of upto 60 sgRNAs, applicable for prime editors	Lower sensitivity with higher multiplexing
	Direct *in situ* breaks labelling, enrichment on streptavidin and next-generation sequencing (BLESS) Crosetto et al. (2013)	Cells are fixed to preserve DSBs, nuclei are isolated and the DSBs are blunted and ligated to a biotinylated linker. gDNA is then isolated and biotinylated sequences are enriched with streptavidin beads and sequenced	Nucleotide-resolution DSB mapping, applicable to tissues derived from *in vivo* studies	Only provides a snapshot of the DSBs present in the cells at the time of fixation, can miss DSBs unless a very large number of cells is profiled. Low signal to noise ratio due to the cell fixation and handling steps, requires high input DNA/cells. Centrifugation steps in the protocol can damage the chromatin and introduce spurious DSBs and are incompatible with smaller nuclei
	Breaks labelling *in situ* and sequencing (BLISS) Yan et al. (2017)	Cells/tissues are fixed and attached to glass slides, and DSBs are labelled with a dsODN with a T7 promoter that serves to amplify the DSB sequences by *in vitro* transcription	More sensitive than BLESS, amenable to multiplexing, lower input requirement than BLESS	Only provides a snapshot of the DSBs present in the cells at the time of fixation, can miss DSBs unless a very large number of cells is profiled
	Surveyor Guschin et al. (2010)	Target DNA from both mutant and wild-type reference DNA are amplified by PCR and hybridized; followed by treatment of annealed DNA with Surveyor endonuclease to cleave heteroduplexes and analysis of digested DNA products	Rapid, relatively simple and cost-effective method	Requires *a priori* knowledge. Lacking in single nucleotide resolution. Cannot discriminate between alleles. Preferentially identifies substitutions
	T7E1 Mashal et al. (1995)	Target DNA from both mutant and wild-type reference DNA are amplified by PCR and hybridized; followed by treatment of annealed DNA with T7E1 endonuclease to cleave heteroduplexes and analysis of digested DNA products	Rapid, relatively simple and cost-effective method	Requires *a priori* knowledge. Lacking in single nucleotide resolution. Cannot discriminate between alleles. Preferentially identifies insertions and deletions
	TIDE, TIDER Brinkman et al. (2014), Brinkman et al. (2018)	PCR amplification of candidate sites followed by Sanger sequencing and bioinformatics analysis to identify off-target events	Provides details about the indels and mutations generated. User-friendly interface. Very affordable	Low throughput. Requires *a priori* knowledge. Requires fine tuning of settings by the user
	LAM-HTGTS Frock et al. (2015)	Genome-wide detection of “prey” DSBs via their translocation to a fixed “bait” DSB in cultured mammalian cells	Very high sensitivity	High input requirement
	PE-tag Liang et al. (2023)	DNA tag integration at target site and off-target sites by prime editor, followed by tagmentation and tag-specific amplification	Rapid and sensitive approach for the genome-wide identification of prime editor activity and evaluation of safety	Sensitivity to an off-target site is limited to sequences that can be extended by the associated reverse transcriptase. Low sensitivity *in vivo* due to modest editing efficiencies of PEs
	Detect-seq Lei et al. (2021)	Chemical labeling of deoxyuridine and biotin pulldown of CBE-edited DNA followed by deep sequencing	Genome-wide identification of CBE-induced off-target sites	Analysis limited to tools that generate deoxyuridine as an editing intermediate
	CAST-seq Turchiano et al. (2021)	PCR amplification uses a “bait primer” binding to the on-target sequence, a “prey primer” that recognizes the linker sequence, and “decoy primers” that bind the target sequence to prevent on-target amplification. Further PCR amplifications are successful only if the binding sites of the decoy primers are lost because of translocations or large deletions at the on-target site	High sensitivity and quantitative measurement of chromosomal rearrangements Can be performed directly in the clinically relevant cell-type	Does not recognize off-target sites that are repaired exclusively by NHEJ, not always is possible to design effective bait and decoy primers for a desired locus
	SURRO-seq Pan et al. (2022)	Targeted in-cell capture of off-targets based on a pooled lentiviral library encoding a sgRNA and barcoded surrogate off-target sites	Higher scalability than previous targeted methods, e.g., TIDE	Targeted approach, requires pre-selection of candidate sites
*in vivo*	DISCOVER-Seq Wienert et al. (2019)	A modified chromatin immunoprecipitation approach where DSBs are indirectly identified as sites bound by meiotic recombination 11 homolog 1 (MRE11), a DNA repair protein that is part of the MRE11-RAD50-NBS1 (MRN) complex that colocalizes to DSBs created by CRISPR-Cas before repair	Can be applied *in vivo*	Lower sensitivity (∼0.3%) and high false positive rate
	GUIDE-Tag Liang et al. (2022)	Modification of GUIDE-seq where Cas9 protein is fused with monomeric streptavidin (mSA), which helps to improve the rate of incorporation into DSB sites of a biotinylated dsODN that is delivered separately	Can be applied *in vivo*, can identify SVs, gDNA library compatible with UdiTas for identifying SVs	Low insertion rate of the dsODN
	VIVO Akcakaya et al. (2018)	*In vitro* discovery of off-targets by CIRCLE-seq Tsai et al. (2017) followed by *in vivo* validation	High sensitivity and applicable to whole organisms	*In vivo* validation is restricted to a subset of candidates
	GOTI Zuo et al. (2019)	Editing of single blastomeres of two-cell mouse embryos and progeny cells are examined by WGS	Suitable for CRISPR-Cas9 and base editors. Only detects edits that are improperly repaired and transmitted to daughter cells, directly compares edited and non-edited cells with identical genetic backgrounds	Results are specific to the species in which it is performed. Very expensive method. Requires high level of technical skill and specific apparatus

## 3 Approaches to reduce off-target genome editing

### 3.1 Improvement of nucleases

The RNA-guided CRISPR-Cas9 nuclease from *Streptococcus pyogenes* (SpCas9) has been extensively repurposed for genome editing. Early studies revealed its high DNA cleavage activity but also associated it with increased off-target events (Hsu et al., 2013; Mali et al., 2013). To address this, SpCas9 mutants with greater specificity were developed, including enhanced SpCas9 (Slaymaker et al., 2016), high-fidelity SpCas9 (Kleinstiver et al., 2016), and hyper-accurate Cas9 (Chen et al., 2017). These variants displayed significantly fewer off-target effects than the original SpCas9. A recent innovation, SuperFi-Cas9 (Bravo et al., 2022), can discriminate between on- and off-target DNA substrates without compromising DNA cleavage, offering high fidelity. However, it exhibits relatively low on-target activity, limiting its general use in gene editing applications (Kulcsar et al., 2022). Nevertheless, in certain scenarios, SuperFi-Cas9 can complement super-active adenine base editors, counteracting mutant deaminase partners. Other lower-activity, higher-fidelity SpCas9 variants, such as HeFSpCas9 (Kulcsar et al., 2017), also enhance the precision of hyperactive editors. While high-fidelity Cas9 variants excel in specificity, they may exhibit reduced on-target activity when delivered as ribonucleoprotein (RNP) complexes, which is a therapeutically relevant formulation. In this context, HiFi Cas9 shows improved on-to-off-target ratio when delivered as an RNP, facilitating robust gene targeting for the correction of disease-causing mutations (Vakulskas et al., 2018). Finally, Cas9 nickases, which cut only one DNA strand, generate DSBs with reduced off-target effects when guided by two sgRNAs (Ran et al., 2013; Frock et al., 2015). However, challenges arise in identifying properly positioned sgRNAs due to the requirement of PAM sequences. While the diversity of genome editing tools continues to expand (Altae-Tran et al., 2021; Saito et al., 2023), complete elimination of off-target effects remains a major challenge in the field.

### 3.2 Improvement of sgRNAs

The design and engineering of sgRNAs are critical factors that can significantly impact the fidelity of genome editing when using CRISPR systems. Genome-wide CRISPR studies have revealed that sgRNAs targeting the same gene locus can yield different results, underscoring the importance of carefully selecting sgRNAs *in silico* (Shalem et al., 2014; Wang et al., 2014; Doench et al., 2016). Beyond sgRNA sequence, modifying its length can enhance Cas9 specificity. This can be done by either extending sgRNAs with the addition of two guanine nucleotides at the 5′ end (Cho et al., 2014) or by truncating sgRNAs by removing 2–3 nucleotides from the 5′ end (Fu et al., 2014). Additionally, modifications like 2′-O-methyl-3′-phosphonoacetate (Ryan et al., 2018), next-generation bridged nucleic acids (Cromwell et al., 2018) and locked nucleic acids (Cromwell et al., 2018), reduce off-target effects while maintaining on-target efficiency. However, these modifications are primarily suitable for sgRNAs delivered as a RNA molecule, but not for sgRNAs encoded as a transgene.

### 3.3 DSB-independent editing

Cas9-mediated DSBs have been a major source of off-target effects in CRISPR-Cas9 genome editing. Emerging gene editing tools aim to sidestep DSBs, thus enhancing specificity. One such innovation is base editors (BEs), which employ Cas9 nickases fused with nucleotide deaminases to induce single nucleotide changes via single-strand breaks (SSBs). There are two primary types: cytosine base editors (CBEs) (Komor et al., 2016), that convert cytosine to thymine, and adenine base editors (ABEs) (Gaudelli et al., 2017), that convert adenine to guanine. Additionally, the development of novel BEs capable of C>G transversions have expanded their utility (Kurt et al., 2021).

Prime editing (PE) represents a groundbreaking advancement as this can accurately create various genomic alterations, including substitutions and small insertions/deletions (Anzalone et al., 2019; Chen et al., 2021; Doman et al., 2023). This technology employs a Cas9 nickase-reverse transcriptase fusion protein and an engineered prime editing guide RNA (pegRNA). Notably, PEs exhibit reduced off-target activity compared to CRISPR-Cas9, enhancing their safety for therapeutic applications, and they have shown promise in treating sickle cell disease by correcting its primary genetic cause (requiring a transversion in HBB) (Anzalone et al., 2019).

Despite BEs and PEs mitigating large-scale genomic changes linked to DSBs, they are not without off-target risks. BEs have been associated with unintended off-target effects, such as RNA off-target activity, including self-editing of BE transcripts (Grunewald et al., 2019) and sgRNA-independent DNA editing (Jin et al., 2019; Zuo et al., 2019). Off-targets effects at the RNA level are likely transient and detectable via RNA-seq, whereas sgRNA-independent editing occurs at sites with exposed single-stranded DNA and is typically assessed by methods based on whole genome sequencing (WGS) (Table 2).

An alternative strategy to minimize off-target genomic edits is through epigenetic editors. These tools, using dCas9 fused with epigenetic modifiers, can modulate endogenous gene expression (Qi et al., 2013; Chavez et al., 2015; Hilton et al., 2015; Thakore et al., 2015; Liu et al., 2016) and they have been employed successfully *in vivo* to treat diseases such as diabetes mellitus, muscular dystrophy, acute kidney injury, obesity, and inherited blindness (Liao et al., 2017; Kemaladewi et al., 2019; Matharu et al., 2019; Bohm et al., 2020). It is important to note that while these tools don’t induce permanent DNA changes, they can impact the epigenome, potentially affecting daughter cells. Methods such as Chromatin Immunoprecipitation (ChIP)-seq, assay for transposase-accessible chromatin (ATAC)-seq, and whole genome bisulfite sequencing (WGBS) can be used to assess epigenetic off-target effects. In summary, advancements in gene editing tools, including BEs, PEs, and epigenetic editors, aim to enhance specificity and reduce off-target effects. However, careful scrutiny and evolving detection methodologies remain essential to ensuring the safety and precision of genome editing applications.

### 3.4 Anti-CRISPR proteins

Controlling CRISPR activity is crucial for developing safe gene-editing therapeutics. CRISPR-inhibitory molecules can serve as a failsafe mechanism to deactivate the CRISPR-Cas complex or enhance precision by decreasing off-target effects. Anti-CRISPR (Acr) proteins, initially discovered in 2013, have evolved in phages to counteract bacterial and archaeal CRISPR systems (Bondy-Denomy et al., 2013). Over 80 naturally occurring Acr proteins have been identified (Jia and Patel, 2021), and one synthetic small molecule inhibitor was discovered through high-throughput screening (Maji et al., 2019). Acr proteins can be co-expressed with Cas9 or fused directly to it, for fine-tuning its activity and improving target specificity (Aschenbrenner et al., 2020). AcrIIA4, for example, reduces off-target effects in human cells by interfering with the DNA recognition ability of Cas9 without compromising on-target gene editing (Shin et al., 2017). Acr proteins have also shown promise in attenuating RNA targeting and editing by Cas13a, thus reducing off-targeting (Lin et al., 2020), and they can suppress base editing, minimizing its off-target effects in mammalian cells (Liang et al., 2020). Innovatively, Acr proteins have been employed to enable conditional optogenetic control of Cas9. AcrIIC3-LOV2 hybrids effectively block Cas9 activity in the absence of light but permit genome editing upon light exposure in human cells (Hoffmann et al., 2021). Finally, inducible hybrids of Acr proteins and 4-hydroxytamoxifen-responsive intein enable post-translational control of CRISPR-mediated genome editing (Song et al., 2022). While these approaches hold promise for precise control of genome editing, they are not without risks, and further research is needed to rule out potential toxic effects or immune responses triggered by Acr proteins in humans.

### 3.5 Improvement of delivery methods for spatiotemporal control of editing

The delivery format of Cas9, whether as DNA, mRNA, or ribonucleoprotein (RNP), plays a crucial role in determining its expression level and exposure duration, impacting both editing efficiency and off-target activity. Short-lived formats like RNP and mRNA have demonstrated lower off-target activity compared to plasmid DNA (Kim et al., 2014; Ramakrishna et al., 2014), aiming for a transient peak expression of CRISPR-Cas9 followed by rapid turnover to prevent off-target effects associated with prolonged expression (Cameron et al., 2017). In the context of *in vivo* genome editing, the duration of gene editor expression becomes a pivotal consideration when selecting delivery vectors. Adeno-associated virus (AAV) vectors, known for their capacity to sustain long-term gene expression, are favored for *in vivo* gene therapy. Self-inactivating Cas9 and AAV delivery vectors have been devised to mitigate prolonged exposure to genome editing tools (Epstein and Schaffer, 2017; Li et al., 2019; Ibraheim et al., 2021). Conversely, lipid nanoparticles (LNPs) are emerging as preferred vectors for *in vivo* gene editing, efficiently delivering Cas9 mRNA and sgRNA and undergoing rapid *in vivo* degradation, ensuring transient gene editor expression (Zuris et al., 2015; Wang et al., 2016; Finn et al., 2018). The goal of this strategy is reducing off-target risks and is currently being tested in clinical trials (Gillmore et al., 2021). Recently, it was demonstrated that shuttle peptides can be used for delivering CRISPR RNPs to mouse lung epithelial cells (Kulhankova et al., 2023). This method achieved persistent DNA editing *in vivo*, while showing fast cargo delivery and rapid peptide turnover. An additional layer of control is provided by regulating CRISPR-Cas expression/activity using split Cas9 (Truong et al., 2015; Zetsche et al., 2015), small chemical molecules (Gonzalez et al., 2014; Davis et al., 2015; Dow et al., 2015), light (Nihongaki et al., 2015), and magnetic nanoparticles (Zhu et al., 2019). These methods offer precise control over the timing and extent of Cas9 expression, enhancing safety and specificity, primarily within *ex vivo* editing workflows where high-efficiency payload delivery is feasible. Selecting the right delivery method and vector for gene editors is vital to achieve desired editing efficiency while minimizing off-target effects, particularly in therapeutic contexts emphasizing safety and precision. Ongoing research and development efforts continue to refine and enhance the delivery of CRISPR-Cas9 tools for diverse gene editing applications.

## 4 Pre-clinical assessment of CRISPR-Cas9 off-target activity within the current regulatory framework

In spite of efforts to improve the specificity of Cas nucleases, off-target editing remains a risk and hence must be assessed during the pre-clinical development of CRISPR-based therapies. Given the relatively recent development of CRISPR-based therapeutics, specific regulatory guidelines on the pre-clinical assessment for this class of therapies do not as yet exist. In the United States, all genome editing medicinal products are considered cellular and gene therapy products, and hence the existing guidelines for such products are also applicable to genome editing therapies (FDA-2012-D-1038) (FDA, 2022). In the European Union, the situation is more complex. While most gene editing products would be covered by the Advanced Therapy Medicinal Products (ATMPs) definition, some could be subject to Genetically Modified Organisms (GMO) regulations and requirements, whereas others could be classified as biologicals or even small molecules (EMA/319248/2020) (Mourby and Morrison, 2020; EMA, 2021). The existing guidelines for such products also apply to genomic editing depending on how they are classified (EMA/CAT/GTWP/671639/2008, EMA/CAT/852602/2018, EMA/CAT/80183/2014) (EMA, 2021; EMA, 2019; EMA, 2018). Nevertheless, two recent reports from EMA on gene editing (EMA/319248/2020, EMA/47066/2018) and a recent update to its existing guidelines (EMA/CAT/GTWP/671639/2008 Rev1. Corr.), as well as draft guidelines being developed by the FDA for CAR-T cells (FDA-2021-D-0404) and gene editing products (FDA-2021-D-0398) can provide some insight into the types of pre-clinical off-target assessments that are expected when filing an Investigational New Drug (IND)/Investigational Medicinal Product Dossier (IMPD) application for a gene editing-based medicine (EMA, 2018; EMA, 2021; EU-IN, 2021; FDA, 2022a; FDA, 2022b). Specifically on the topic of off-target editing, regulatory agencies recognize that the existing methods for off-target detection have certain limitations with regards to their sensitivity and specificity. Hence, the use of multiple orthogonal methods, including *in silico*, *in vitro*, and cell-based approaches to assess off-targets is encouraged, and the sensitivity and specificity of these methods should be reported. Nonetheless, the use of an unbiased genome-wide method to evaluate off-targets is considered a key element. Once identified, the potential off-targets must be validated. Methods used for the verification of *bona fide* off-targets should be sensitive enough to detect low frequency events and should be performed in models that are predictable, for example, in the target cell-type. In order to account for natural human genetic variation which can influence off-targeting, such analyses should ideally be performed in cell lines/models from multiple donors. The importance of modelling human genetic variation when assessing off-targeting is discussed in further detail below. Owing to the recognition that Cas9-induced DSBs can generate SVs, methods that can also assess genomic integrity should be included during pre-clinical testing. The biological/physiological consequence of any off-targets identified should also be assessed as feasible, whether in relevant animal models or *in vitro* models. Until there is a greater standardization of methods for the measurement of off-target editing, developers of such products will have to define the strategy for off-target assessment on a case-by-case basis and to consult with the relevant health authorities at an early stage.

Pre-clinical off-target assessment data in combination with clinical data from CRISPR-based therapeutics currently can inform on the choice of the most predictive strategies to assess human off-target editing risk. However, since most CRISPR-Cas9-based therapies are still in the early stages of clinical development, pre-clinical data is often not publicly disclosed and their predictive value for patient safety is not fully known (as of the article’s revision, the public assessment reports for the approval of exa-cel by the regulatory agencies in the United States and the United Kingdom were not yet accessible). Nevertheless, a review of the published pre-clinical studies in support of ongoing clinical trials is instructive. Although different methods have been employed to assess the off-targeting potential of the molecules currently in clinical trials, a common theme is to perform the off-target assessment in two phases: a discovery phase and a validation phase. The discovery phase typically consists of *in silico* or unbiased experimental approaches to identify potential off-targets, whereas the validation phase confirms *bona fide* off-targets among either a subset or all the off-targets identified in the discovery phase in appropriate models. For example, for the molecule NTLA-2001, an SpCas9 mRNA targeting the transthyretin gene via a single sgRNA for the treatment of transthyretin amyloidosis and being developed by Intellia and Regeneron, the discovery phase consisted of three orthogonal methods—*in silico* off-target prediction with Cas-OFFinder, cell-based GUIDE-Seq in HEK293 cells, and the *in vitro* SITE-Seq assay with gDNA from human peripheral blood mononuclear cells (PBMCs) (Gillmore et al., 2021). In the validation phase, all 657 potential off-target sites identified by these three methods were then assessed using rhAMPseq (Dobosy et al., 2011) and amplicon sequencing in primary hepatocytes from two donors at dose levels 27 times the EC90 of the molecule. Of the seven off-target sites that were validated, five were intergenic and two were intronic. Further analysis in a dose-response experiment demonstrated that these off-targets were undetectable at therapeutic doses. Potential SVs introduced by Cas9 cleavage were also assessed using two independent methods—long-range PCR around the TTR locus followed by long-read PacBio sequencing as well as a bespoke SV characterization assay based on short-read sequencing (Gillmore et al., 2021). SVs were identified at low frequencies (<1%) and were considered to be of low risk. A similar approach was employed for assessing off-targeting risk for EDIT-101, a SaCas9 based molecule with 2 sgRNAs that was being developed by Editas Medicine for the treatment of Leber Congenital Amaurosis (Maeder et al., 2019). The discovery phase of the assessment consisted of three orthogonal methods—*in silico* with Cas-OFFinder, GUIDE-Seq in three different cell lines (U2 OS, ARPE19, and SH-SY5Y) and in primary CD4^+^ T cells and fibroblasts, and Digenome-seq. All 145 sites identified, most of which were identified only *in silico*, were then assessed in a validation phase using targeted NGS in U2 OS cells, ARPE-19 cells as well as retinal explants from 2 donors. None of the 145 off-target sites were confirmed in this verification step. Interestingly, GUIDE-seq was additionally used in a preliminary screen in U2 OS cells to identify sgRNAs without any off-targets, which may explain the lack of any validated off-targets. Furthermore, on-target editing efficiency was assessed in human photoreceptors using UdiTas (Maeder et al., 2019).

Such a two-step approach has also been used to assess off-target editing for *ex vivo* therapies. Indeed, for exa-cel, the discovery phase consisted of a combination of *in silico* assessment and GUIDE-seq in hematopoietic stem and progenitor cells (HSPCs) from 3 different donors. All 223 potential off-target sites identified by these 2 approaches were then tested by hybrid-capture followed by NGS in HSPCs from four donors, and none of the off-targets were found above the threshold of detection (Frangoul et al., 2021). Interestingly, when using a variant-aware *in silico* approach to assess the off-targets of the very same gRNA, Cancellieri et al. (2023) were able to identify and validate *CPS1* as a *bona fide* off-target in individuals who were carriers of the alternative allele (C) at single nucleotide variant (SNV) rs114518452. This off-target, although identified *in silico* by Frangoul et al. (2021), was not confirmed in cellular assays due to the lack of representation of rs114518452-C carriers in the donor cells used for the cell-based confirmation assays. Due to the population frequency of this SNV (∼4.5% in African-American populations, and ∼0.01% in Europeans) and the higher prevalence of sickle cell disease in African-American populations, this finding and its implications for the off-targeting consequences of exa-cel were discussed in detail at a FDA Advisory Committee meeting. Although the committee concluded that the risk-benefit profile of the molecule was favorable in spite of this off-target, these discussions highlight the importance of accounting for human genetic variation when assessing off-targeting of CRISPR-based medicines and may portend closer scrutiny of the algorithms and model systems used to investigate this in the future.

In some cases, alternative strategies have also been used for off-target assessment. For example, Stadtmauer et al. (2020) assessed off-target editing in their CRISPR-engineered CAR T-cells using a single unbiased assay, iGuide, at the end of manufacturing. The authors identified a few low frequency off-target edits associated with the different sgRNAs used to knock-out the endogenous T-cell receptor genes (TRAC and TRBC) and immune checkpoint inhibitor (PD-1), yet most of the off-targets were within genes of unknown function or the disruption of whose function was not thought to impact cellular function. However, they did confirm that these edits did not lead to cellular transformation. Due to the propensity for chromosomal rearrangements in the presence of 3 independent sgRNAs, they also assessed translocations at the targeted cut sites using quantitative PCR (qPCR). Although they did identify translocations, especially between the TRAC and TRBC loci, the frequency of translocations diminished over time, suggesting that they do not confer any growth advantage to the cells (Stadtmauer et al., 2020). While there have been no reports of adverse events linked to off-target effects in ongoing clinical trials, it remains premature to ascertain the predictive value of these methods in assessing clinical risks. Indeed, long-term follow-up of patients enrolled in clinical trials will be necessary to determine the impact if any of CRISPR off-target editing on patient health.

## 5 Discussion

Evaluating unintended gene modifications is a pivotal aspect of the pre-clinical development of CRISPR-Cas9-based therapies due to recognized risks tied to such events. Due to the relative novelty of this technology, the ideal screening paradigm to assess off-target editing is still undefined; however, it is likely to employ a range of complementary methods as each method is limited by its inherent sensitivity, as evidenced by certain off-target sites that were missed by other methods (Tsai et al., 2015; Kim et al., 2016; Tsai et al., 2017; Yan et al., 2017; Wienert et al., 2019). A strong collaboration between academia, industry and health authorities is vital to create and harmonize regulations that ensure the safe development of CRISPR-Cas9-based therapies. While ongoing clinical trials have not reported any adverse events linked to CRISPR off-target effects, it’s vital to acknowledge that the long-term impact of these effects on patient health may not manifest immediately. Consequently, continuous monitoring and prolonged patient follow-up are essential to comprehensively grasp the clinical implications of off-target gene editing and to refine safety assessment strategies for forthcoming genome editing-based therapies.
